# Repair of Long Peripheral Nerve Defects in Sheep: A Translational Model for Nerve Regeneration

**DOI:** 10.3390/ijms24021333

**Published:** 2023-01-10

**Authors:** Estefanía Contreras, Sara Traserra, Sara Bolívar, Joaquím Forés, Eduard Jose-Cunilleras, Ignacio Delgado-Martínez, Félix García, Esther Udina, Xavier Navarro

**Affiliations:** 1 Institute of Neurosciences, Department Cell Biology, Physiology and Immunology, Universitat Autònoma de Barcelona, 08193 Bellaterra, Spain; 2 Integral Service for Laboratory Animals (SIAL), Faculty of Veterinary, Universitat Autònoma de Barcelona, 08193 Bellaterra, Spain; 3 Centro de Investigación Biomédica en Red Sobre Enfermedades Neurodegenerativas (CIBERNED), 28031 Madrid, Spain; 4 Hand and Peripheral Nerve Unit, Hospital Clínic i Provincial, Universitat de Barcelona, 08036 Barcelona, Spain; 5 Department of Animal Medicine and Surgery, Universitat Autònoma de Barcelona, 08193 Bellaterra, Spain

**Keywords:** large animal model, long gap, nerve injury, nerve regeneration, sheep

## Abstract

Despite advances in microsurgery, full functional recovery of severe peripheral nerve injuries is not commonly attained. The sheep appears as a good preclinical model since it presents nerves with similar characteristics to humans. In this study, we induced 5 or 7 cm resection in the peroneal nerve and repaired with an autograft. Functional evaluation was performed monthly. Electromyographic and ultrasound tests were performed at 6.5 and 9 months postoperation (mpo). No significant differences were found between groups with respect to functional tests, although slow improvements were seen from 5 mpo. Electrophysiological tests showed compound muscle action potentials (CMAP) of small amplitude at 6.5 mpo that increased at 9 mpo, although they were significantly lower than the contralateral side. Ultrasound tests showed significantly reduced size of tibialis anterior (TA) muscle at 6.5 mpo and partially recovered size at 9 mpo. Histological evaluation of the grafts showed good axonal regeneration in all except one sheep from autograft 7 cm (AG7) group, while distal to the graft there was a higher number of axons than in control nerves. The results indicate that sheep nerve repair is a useful model for investigating long-gap peripheral nerve injuries.

## 1. Introduction

Peripheral nerve injuries result in partial or total loss of motor, sensory, and autonomic functions of the affected nerve territory [1]. When the axon is transected, the segment distal to the lesion disconnects from the neuronal body and undergoes Wallerian degeneration [1,2,3,4] to create a permissive environment for regeneration. In parallel, the axotomized neuron switches to a pro-regenerative state, with phenotypic changes to support axon re-growth [5]. Despite adult peripheral neurons having this intrinsic regenerative capability and being able to reinnervate their target organs eventually, functional outcome is not always complete [1,6,7], leading to chronic functional impairments and decreased patient quality of life.

The severity of the nerve injury is one of the main factors that determines the degree of recovery. Therefore, mild injuries, such as compression or crush, in which only the axons are transected but all the connective layers, including the endoneurial tubules, are preserved, usually recover normal function. In contrast, when both axons and connective tissue are transected, functional outcomes are worse. After a complete nerve transection, surgical repair is mandatory to suture the proximal and distal nerve stumps together and, thus, facilitate that axons can cross the gap and grow along the distal stump. When direct suture of the two stumps is not possible because the separation is too large, a bridge is required to guarantee continuity between the two nerve stumps. Currently, the gold standard for this type of lesion is the interposition of a segment of another nerve from the same patient, called an autologous graft [6,8]. However, long nerve defects cannot always be repaired with an autograft due to the limited availability of nerve sources for the repair of extensive or multiple nerve injuries. In addition, autograft harvesting can lead to neuroma formation and permanent loss of sensation in the territory of the donor nerve [9].

Thus, regeneration of long nerve defects remains a challenge in the clinic, and further research is needed to find alternatives to autograft repair. Tube repair, the implantation of a tube or conduit from natural or synthetic biomaterials to bridge a nerve gap, emerged as a potential alternative to the autograft repair of transected peripheral nerves [10]. However, depending on the size of the injured nerve and the species, the regeneration across nerve conduits is limited by the length of the gap. Experimental studies showed that regenerating axons can bridge empty tubes made of silicone or synthetic materials along a gap of up to 4 mm in the mouse [11], up to 10 mm in the rat [10], and 30 mm in primates [12,13], but fail in most cases with longer gaps. Therefore, the most relevant clinical need for alternative repair methods in human patients are lesions resulting in gaps between 3–30 cm in length [14].

The rat sciatic nerve transection and repair model is the most widely used rodent model in peripheral nerve regeneration [15,16,17,18,19]. Nerve anatomy of rodents has been well studied, but regeneration is faster than in humans and, obviously, only short nerve gaps can be studied [17,20,21]. In addition, the recovery of limb function may be better because of the short regeneration distances between the injured nerve and the target organs in rodents [22]. It is fundamental to provide good experimental models with the aim to explore and design new and innovative therapies with translational potential.

Large animal models allow for longer gaps and longer regeneration distances that mimic the clinical conditions often found in human nerves. The development of new clinical approaches in peripheral nerve regeneration includes preclinical animal testing in animal models that reproduce the regeneration process that occurs in human nerve injuries [17]. There is no standard large animal model for nerve repair studies [9]. The large animals used for peripheral nerve injury and regeneration studies include primates, dogs, cats, pigs, and sheep [17,23,24,25]. The sheep has gained interest as one of the most relevant animal models for preclinical studies [25,26,27,28]. Compared to other large animals, sheep have advantages because of their availability, simplicity of care and housing, cost, and social acceptance [20,29,30]. They have generated interest for the study of long nerve gap repair because their peripheral nerves resemble human nerves in terms of their length, diameter, and function [9,20,31]. Protocols for nerve surgery and the clinical evaluation of deficits and histological processing have been recently proposed [25].

The aim of this study is to standardize a model of peripheral nerve injury in sheep and adequate methods for evaluation during a long follow-up period that will be useful for the investigation of new therapeutic alternatives to the autograft for the repair of severe, long-gap nerve injuries. We include the surgical approach, functional monitoring, electrophysiological and ultrasound tests, and histological analyses, for a comprehensive and detailed quantification of nerve regeneration and reinnervation.

## 2. Results

### 2.1. Clinical Observations

The surgical approach allowed the dissection of the peroneal nerve over a long distance, its resection, and its repair by interposing the same nerve segment with either a 5 or a 7 cm autograft, AG5 and AG7, respectively. All the sheep recovered well from the surgery and survived to the end of the study. In addition, no significant clinical signs were observed during the experimental study. After the surgery, animals were able to stand and walk and showed good mobility. As a result of the peroneal nerve injury, two animals from AG7 group had a marked foot drop posture and developed pressure ulcers. A molded plastic splint was placed at 1 month postoperation (mpo), and the skin ulcers were treated with chlorexhydine and Blastoestimulin cream and covered with a Vetrap bandage. After one month, one of the sheep recovered and the splint was removed. In contrast, the other sheep continued with the foot drop posture until the end of the study.

### 2.2. Functional Evaluation

**Figure 1:**
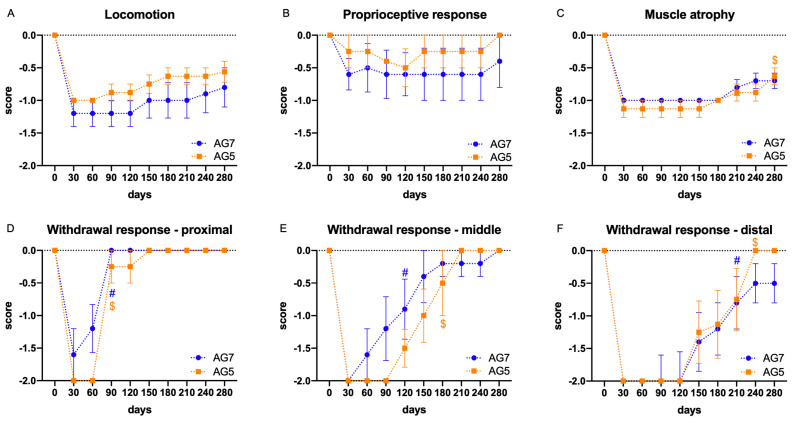
Plots of the monthly functional tests during the follow-up (9 months) in the AG5 and AG7 groups. Results are expressed as mean ± SEM. (**A**): Locomotion, scored based on the occurrence of foot drop during fast walking, was reduced and did not have significant recovery. (**B**): The proprioceptive response was slightly impaired and did not show noticeable changes. (**C**): The mass of the TA muscle was reduced postinjury and significantly recovered ($ *p* < 0.01) vs. baseline at 30 days postoperation, at the end of the follow-up in AG5 group. (**D**–**F**): The withdrawal response to pinching the skin of the dorsum of the foot recovered to close to normal levels in AG5 group. (**D**): The responses recovered significantly in AG5 and AG7 groups at 90 days postoperation ($ *p* < 0.0001 and # *p* < 0.0001 vs. baseline at 30 days postoperation, respectively) in the proximal site. (**E**): In the middle site, recovery was observed at 120 days postoperation in the AG7 group (# *p* < 0.05) and at 180 days postoperation in the AG5 group ($ *p* < 0.01), compared to baseline at 30 days postoperation. In both cases, the responses were earlier than in the distal site, likely due to collateral reinnervation. (**F**): Pinching the distal site showed a response compatible with peroneal reinnervation, with significant improvement at 210 days postoperation in AG7 group (# *p* < 0.05) and at 240 days postoperation in AG5 group ($ *p* < 0.001) vs. baseline at 30 days postoperation.

After the surgery, all sheep showed deficit in locomotion score based on the occurrence of foot drop during fast walking. In the resting orthostatic position, all the sheep, except the two animals of AG7 group indicated above, were able to maintain the plantar support of the right hindlimb. In all the sheep, we observed evidence of foot drop during fast walking, as they failed to maintain the plantar support in some steps (scored as −1 or −2) (Figure 1A). The proprioceptive response was not significantly reduced after the surgery and did not change during the follow-up (Figure 1B). The muscle mass of the right reinnervated tibialis anterior (TA) muscle showed a clear reduction one month after the surgery in comparison to the contralateral muscle. A significant improvement (*p* < 0.01) was detected in AG5 group at the end of the follow-up with respect to values at 30 days (Figure 1C). The withdrawal reflex response to pinching the skin of the dorsum of the foot was abolished at the first test after the surgery and recovered slowly during the follow-up to close to normal levels, early in proximal (*p* < 0.0001) (Figure 1D) and middle (*p* < 0.05) (Figure 1E) sites, likely due to collateral reinnervation. In contrast, the reflex in the distal site showed a return of response compatible with peroneal reinnervation, reaching full recovery in all the sheep of AG5 group at the end of the study (*p* < 0.001 vs. values at 30 days), and in all except one in the AG7 group (*p* < 0.05 vs. values at 30 days) (Figure 1F).

### 2.3. Electrophysiological Results

**Figure 2:**
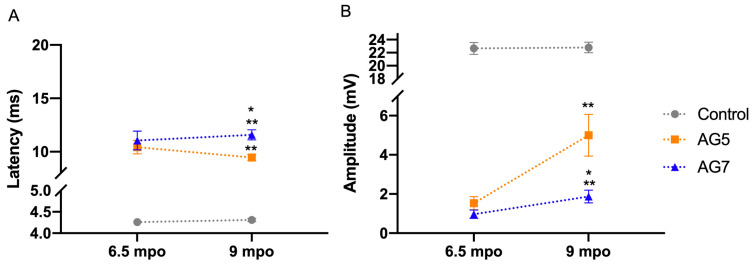
Plots of the latency (**A**) and the amplitude (**B**) of the TA CMAP evoked by stimulation of the sciatic nerve at the sciatic notch and recorded at 6.5 and 9 months (mpo) after surgical insertion of autografts of 5 and 7 cm length, AG5 and AG7, respectively, and compared with the measurements made in the control contralateral hindlimb. (**A**). Small and long latency CMAPs were recorded in 3 of the sheep of each experimental group at 6.5 mpo. (**B**). The CMAP amplitudes increased at 9 mpo, although they were much lower than in control muscles. Values are represented as mean ± SEM. * *p* < 0.05 AG7 vs. group AG5; ** *p* < 0.0001 AG5 and AG7 groups vs. Control.

Motor nerve conduction tests were performed at 6.5 and 9 months after the surgery under general anesthesia (diazepam 0.25 mg/kg and ketamine 5 mg/kg i.v.), to assess reinnervation of TA muscle with an electromyography (EMG) apparatus (Sapphire 4ME, Vickers Healthcare Co., Surrey, UK). In the left control hindlimb, the TA compound muscle action potential (CMAP), evoked by stimulation of the sciatic nerve at the sciatic notch, appeared at an average of 4.3 ± 0.1 ms of latency and had a mean amplitude of 21.2 ± 0.7 mV considering the nine sheep of the study (Figure 2). In the right, operated hindlimb, at 6.5 mpo, 75% of the animals from AG5 group and 60% of the animals from AG7 showed consistent evidence of reinnervation with CMAPs at long latency, with disperse shape and small amplitude (1.53 ± 0.67 mV in group AG5 and 0.97 ± 0.48 mV in group AG7). At 9 mpo, all the sheep of group AG5 but only 80% of group AG7 group had a positive CMAP. The mean CMAP amplitude of group AG7 (1.87 ± 0.72 mV) was significantly lower than in group AG5 (5.00 ± 2.13 mV) (Figure 2A), and the onset latency was significantly longer in AG7 group (11.58 ± 1.06 ms) compared to AG5 group (9.46 ± 0.6 ms) (Figure 2B).

### 2.4. Echographic Evaluation of TA Muscle

**Figure 3:**
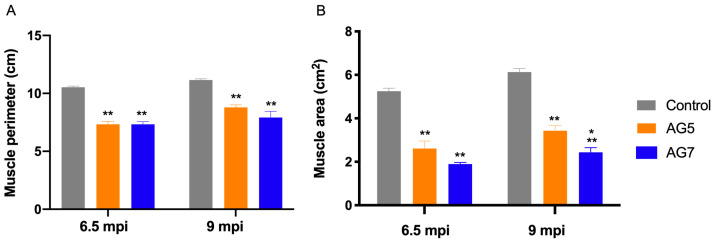
Histogram of the measurements of perimeter (**A**) and area (**B**) of the TA muscle obtained by echographical imaging. Values are mean ± SEM. * *p* < 0.05 group AG7 vs. AG5; ** *p* < 0.0001 groups AG7 and AG5 vs. Control.

Echography of TA muscle was performed following the electrophysiological tests, at 6.5 and 9 mpo, under general anesthesia. In the operated hindlimb, the size of the TA muscle was significantly decreased, and the echo density changed due to muscle atrophy secondary to denervation. At 9 mpo, the muscle size, the perimeter (Figure 3A), and the area (Figure 3B) were still below control values, although the muscle area was significantly lower (*p* < 0.5) in the group AG7 (2.44 ± 0.21 cm^2^) compared to the group AG5 (3.43 ± 0.23 cm^2^) (Figure 3B). The TA muscle size of the sheep without EMG recovery was the smallest, linking muscle size to degree of reinnervation. The TA muscle was weighed fresh after extraction. The mean values in the experimental sheep of AG5 and AG7 were 38.5 ± 1.4 g and 33.0 ± 4.5 g, and were significantly lower (*p* < 0.05) than 78.8 ± 4.8 g for the left control muscles.

### 2.5. Histological Results of the Grafted Nerve

**Figure 4:**
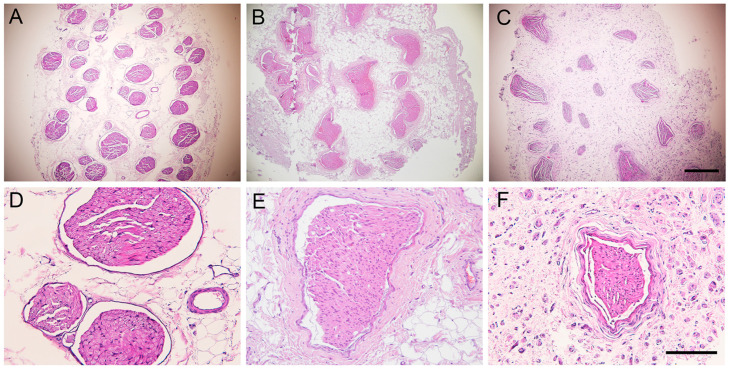
Representative micrographs of cross-sections of the middle part of the nerve graft stained with hematoxylin and eosin. (**A**,**D**) control nerve, (**B**,**E**) nerve of a sheep of group AG5, and (**C**,**F**) of group AG7, viewed at 40× magnification (**A**–**C**), scale bar 500 μm, and at ×200 magnification (**D**–**F**), scale bar 150 μm.

After harvesting, the nerve autografts of all the sheep showed neuromata that were visible at both proximal and distal suture lines, but the autograft had a well-preserved appearance. The sheep peroneal nerve is composed of multiple fascicles, usually more than 30, each containing numerous nerve fibers densely packed in the endoneurium. The fascicular structure was maintained in the autografted segment in both experimental groups. Regenerative axons were seen both inside and outside the nerve fascicles (Figure 4).

**Figure 5:**
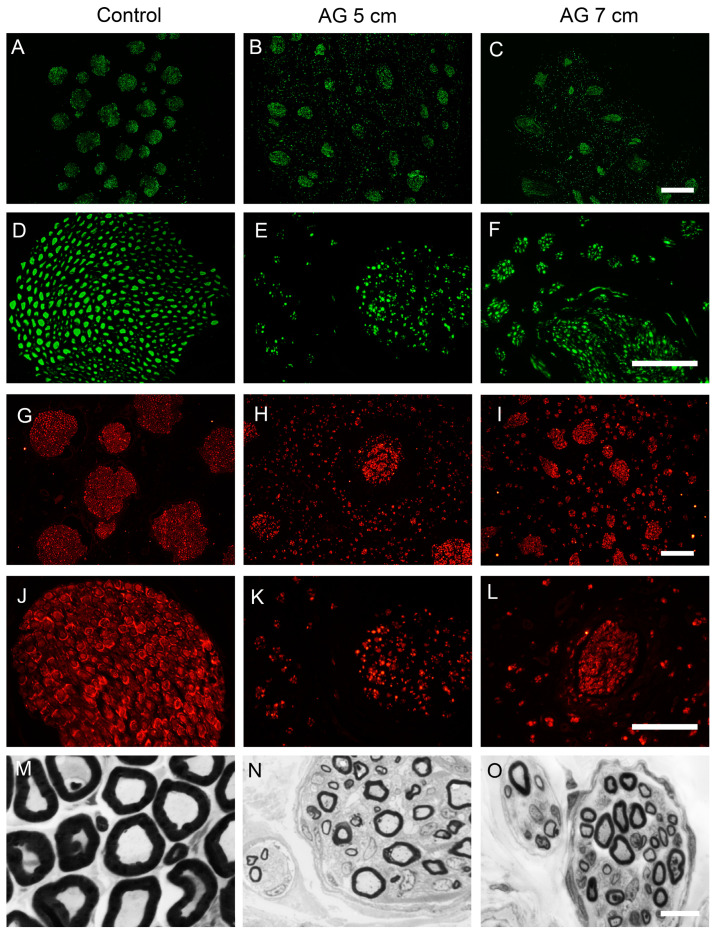
Representative immunohistochemical images of transverse sections of a control peroneal nerve (**A**,**D**,**G**,**J**) and of an autograft of group AG5 (**B**,**E**,**H**,**K**) and of group AG7 (**C**,**F**,**I**,**L**). Sections were immunolabeled against NF200 for myelinated axons (**A**–**F**), and against S100 for Schwann cells (**G**–**L**). Images were taken at ×40 magnification (**A**–**C**,**G**–**I**), scale bar 200 μm, and at ×400 magnification (**D**–**F**,**J**–**L**), scale bar 100 μm. The bottom panels show representative semithin transverse sections of the middle segment of the nerve graft stained with toluidine blue. (**M**) control nerve, (**N**) AG5, and (**O**) AG7 graft, at 10,000× magnification, scale bar 10 μm.

Immunohistochemical labeling for NF200 and S100 in sections taken at the middle of the nerve grafts showed the presence of myelinated axons and Schwann cells in the intrafascicular and extrafascicular space of the autografts. In contrast, distal to the nerve autograft, myelinated axons and Schwann cells were only observed within the fascicles (Figure 5). In semithin transverse sections of the mid segment of the nerve autograft of both experimental groups, we found that myelinated axons were grouped in small regenerative units and were distributed throughout the autograft structure, as well as unmyelinated axons and Schwann cells (Figure 5M–O).

Regarding the quantitative analysis of NF200 positive myelinated axons, one animal of group AG7 did not show regenerated axons, likely attributable to suture dehiscence in early phases after the surgery. The estimated mean number of myelinated axons in the control peroneal nerve was 18,022 ± 2040 axons. At the mid-level of the autograft, there was a significantly higher number of myelinated axons in the AG5 group compared to group AG7 (57,294 ± 1368 axons and 26,213 ± 2798 axons, respectively, *** *p* < 0.001). Distal to the nerve graft, there were also significantly more axons in group AG5 (36,185 ± 3533 axons) compared to group AG7 (20,600 ± 6082 axons) (*** *p* < 0.001).

### 2.6. Histological Evaluation of Reinnervated Targets

**Figure 6:**
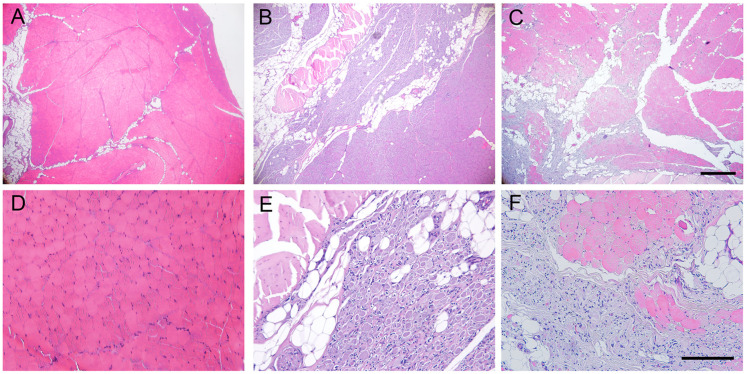
Representative micrographs of cross-sections of the tibialis anterior muscle stained with hematoxylin and eosin from (**A**,**D**) a control muscle, (**B**,**E**) a sheep of group AG5, (**C**,**F**) a sheep of group AG7, taken at ×40 magnification (**A**–**C**), scale bar 200 μm, and at ×200 magnification (**E**–**F**), scale bar 100 μm. Note the areas of hypotrophic muscle fibers with inflammatory cell infiltration in the denervated muscle areas of AG sheep.

The intact TA muscle showed an organized structure of elongated muscle fibers with the nuclei at the periphery. In cross-sections, histological images showed compact muscle fibers, polygonal in shape and surrounded by basal lamina (Figure 6A). In the operated hindlimb, the TA muscle showed an irregular structure in both experimental groups. Some areas appeared with a normal structure, although the muscle fibers had smaller diameter than normal, likely corresponding to reinnervated areas of the muscle, whereas other areas showed signs of atrophy and inflammatory cell infiltration (Figure 6). The skin samples of control hindlimb, in sagittal sections, showed distinct organization in three layers: epidermis, dermis, and subcutaneous tissue. The skin samples of the operated hindlimb showed a similar aspect to the skin from the contralateral side, without signs of inflammatory cell infiltration or tissue atrophy.

## 3. Discussion

In this study, we have shown that nerve regeneration is successful after autograft repair of a large defect in the peripheral nerve of sheep and can be objectively evaluated. Thus, we propose that the sheep is a suitable model to evaluate regeneration through long nerve gaps. Sheep represent an adequate large animal model because they have similar body weight and peripheral nerve dimensions to humans. Unlike rodent species, sheep have plurifasciculated nerves [32] and a regeneration rate identical to humans [33,34]. Compared to other large animals used, including pigs, sheep are calm, easy to obtain, and cost-effective, and they allow the evaluation of sensory and motor functions with the same methods used in the clinic. In addition, their life expectancy is sufficiently long to allow long-term studies to compare with studies of recovery after human peripheral nerve injuries, requiring at least 2 years for recovery to plateau values [35,36,37]. Other useful models in preclinical studies, like conventional pigs, would be unusable for long-term studies due to their high growth rate, in addition to their stressful and nervous behavior. Other large species, which include nonhuman primates and dogs, present more ethical concerns.

For nerve injury in an animal model to be a useful model with translational potential, the injured nerve must be also relevant. In humans, the most frequently injured nerve in the lower limb is the common peroneal nerve [25,38]. In sheep, this nerve is similar in size to the human nerve [34,37] and it is also plurifascicular, giving rise to the deep peroneal nerve branch that innervates muscles in the anterior compartment of the hindleg, and to the superficial peroneal nerve providing sensory innervation to the dorsum of the paw. Surgical access to the peroneal nerve is an easy procedure with blunt dissection of the semitendinosus and biceps femoris muscles. Since the common peroneal nerve is a mixed nerve, both motor and sensory functional losses in the denervated targets are expected [39]. The injury of this nerve in sheep mimics the symptoms of the inversion of the foot and inability to dorsiflex the ankle [25,40]. However, the motor deficits are limited, and do not markedly disturb standing and walking of the animals [32,37]. We observed that the sheep did not show disabling consequences of the nerve surgeries in their locomotor motion or a reduced quality of life. This was so despite two animals showing a sustained foot drop position that, together with the lack of sensitivity in the dorsum of the foot, led to focal pressure ulcerations.

Other studies producing a transection and relatively long gaps in the sheep used the median nerve in the forelimb, with gaps ranging from 5 mm to 5 cm [22,24,30,34,41,42], or the facial nerve inducing just transection or gaps up to 5 cm [27,43]. However, injuries of the forelimb nerves lead to more functional deficits, since compensation for animal weight support and movement is more effective in the hindlimbs [37]. The sciatic and the tibial nerves have also been subjected to gap lesions and repair with different conduits bridging a 1 cm gap [44,45,46], and with recellularized allografts in a 2 cm gap [47] in comparison with autologous nerve grafts. The sheep peroneal nerve has been used in a few studies prior to the present study. Strasberg and colleagues [32] compared the outcomes of the surgical repair of the peroneal nerve via insertions of 8 cm long nerve autografts and allografts. Histological and electrophysiologic analyses were carried out at 6 and 10 months. Roballo et al. [37] compared electrophysiological and histological outcomes after peroneal nerve transection and a 5 cm autograft in adult sheep. Alvites et al [25] compared only functional outcomes 3 and 6 months after peroneal transection and repair by direct suture or via a chitosan conduit leaving a ~24 mm gap. In the study by Tamez-Mata et al. [48], a peroneal nerve segment of 30 mm in length was excised, and repair was performed by an autograft or an allograft recellularized with Schwann-like cells. Altogether, the peroneal nerve in sheep is a good model for preclinical trials in which several-centimeter-long gaps can be repaired with newly developed grafts or synthetic conduits in comparison with the standard autologous graft.

Importantly, the long distance that axons must grow in the sheep hindlimb requires a considerably long follow-up for assessment of reinnervation of distal targets and meaningful functional recovery. We measured the distance between the proximal section of the peroneal nerve and the entrance of the distal nerve into the TA muscle, and it was between 34–36 cm. Considering a rate of regeneration of 1–2 mm/day, similar to humans, 6 to 12 months would be needed for regaining muscle innervation, and even longer for reinnervation of the dorsum of the hindfoot. Our evidence of TA muscle reinnervation by nerve conduction tests at 6.5 months suggests a regeneration rate ~2 mm/day, in line with previous results reported by Strasberg et al. [32], Radtke et al. [45], and Roballo et al. [37], who performed similar electrophysiological tests. Indeed, an average axonal regeneration velocity of 1.57 mm/day was estimated in sheep that received an autologous nerve graft in the tibial nerve [46]. It is worth noting that the time courses of functional recovery for surgical repair of both 5 and 7 cm lengths of the autografts used to repair the transected peroneal nerve were parallel, even though the functional recoveries were less when 7 cm long autografts rather than 5 cm long grafts were inserted. These observations indicate that the length of the autograft plays a role.

We used clinical evaluations for assessing functional recovery of sensory-motor functions after autograft repair in sheep that are used regularly in human patients. These quantifiable functional behavioral measures provided important indicators of recovery that were complemented by electrophysiological measures. We established monthly intervals for a functional evaluation to assess the deficits produced by the peroneal nerve injury and the recovery course. Peroneal nerve injury, as in humans, results in inability to dorsiflex the ankle [25,40]. In the standing position, most sheep were able to correctly place the hoof and make plantar contact with the ground; however, two of the sheep were unable to do so and showed persistent contact with the dorsum of the foot that led to skin lesions. Whilst we do not know why some sheep presented more marked motor deficits, mechanical factors may be involved. Alvites et al. [25] reported overextension of the hock and overflexion of the distal joints in their sheep, with only slight improvement from 10–12 weeks after neurotmesis and direct suture repair. Testing foot placement during open locomotion in the barn allowed us to detect foot drop in the operated paw in all the sheep. The incidence of foot drop partially improved in two sheep of each group from 5 mpo onwards, but the mean score of the groups did not change significantly during follow-up. That the proprioceptive response in the animal’s hoof was only diminished slightly after the injury may be accounted for by the proprioception conveyed by the tibial and sural nerves that remained intact. Evaluating the time to response using a similar maneuver, Alvites et al. [25] found a small reduction in time but already at 6–8 weeks after direct suture. Therefore, measurement of the proprioceptive response has limited use in this model. A fast recovery of the withdrawal reflex induced by painful stimulation in the peroneal cutaneous territory, was observed at early times in proximal and mid sites of the dorsum of the foot, as reported also by Alvites et al. [25]. This may be explained by reinnervation of the foot by collateral sprouting of neighbor intact nerves that branch from tibial and sural nerves. For this test, the distal third appears to be the one sensitive to peroneal nerve regeneration (see Figure 1F).

Electrophysiological tests are commonly used to objectively evaluate nerve regeneration and muscle reinnervation after nerve injuries in humans and in animals [49,50]. In contrast to small animal models, large animal models allow for easier access to stimulation and recording sites, limiting electrical artifacts [51]. Some animals from both experimental groups showed recordable CMAPs at 6.5 mpo, indicating that regenerating axons had crossed the nerve autograft and into the denervated distal nerve stump to reinnervate the TA muscle. At 9 months, all sheep but one showed positive CMAPs with increasing amplitude and decreasing latency, indicating regeneration and myelination of motor axons. Based on our electrophysiological findings, 9 months appeared to be the minimum timepoint to reliably evaluate different therapies after long nerve gap neurotmesis in the sheep, in agreement with other studies [32,37,45], and the amplitude of the CMAP is the most valuable parameter [50]. Complementing electrophysiology, we used high-resolution echography to evaluate the degree of atrophy of the TA muscle as an indirect measure of reinnervation. This is the first time that ultrasound has been used to quantitatively assess muscle mass after nerve injury in experimental studies.

When the nerve samples were harvested, a small amount of fibrosis was observed around the suture lines in both experimental groups, whereas the diameter of the autografts and the host nerve stumps were similar, contrary to observations when the autograft was from a smaller nerve [51]. In both experimental groups, the peroneal nerve fascicles were preserved but they were slightly smaller than in the control nerve. Myelinated axons and Schwann cells were seen within but also some outside of the fascicles, as commonly reported after neurotmesis [24]. This can be explained by the difficulty of surgically aligning the two ends of the autograft and the proximal and distal stumps of the host nerve. As a result, regenerating axons are likely to be misdirected into pathways that they were not in previously, that in turn, likely result in poor recovery [52,53]. In both experimental groups, myelinated axons of smaller size than normal were seen both within the nerve graft and distal to the graft. The higher number of axons counted compared to the control nerve can be explained by the well-known phenomenon of multiple regenerative sprouts emitted by each axon [54] that, despite a tendency to reduce with time, persist even months after injury in the distal stump [55].

In conclusion, sheep provide an excellent opportunity to study peripheral nerve regeneration in long-nerve gap injuries allowing assays of new repair strategies for the translation towards clinical treatments of nerve injuries. This large animal model addresses key factors for assessing regeneration and can be adequately evaluated by functional tests in addition to electrophysiological and ultrasound tests, as shown in this study.

## 4. Materials and Methods

### 4.1. Animals and Study Design

Nine adult, female ripollesa sheep (Ovis aries) were used. Their age was 2–5 years and their body weight was 55–69 kg. They were obtained from Servei de Granges i Camps Experimentals (SGiCE-UAB, Bellaterra, Spain) of the Universitat Autònoma de Barcelona (UAB, Bellaterra, Spain). The animals were housed in groups in a conventional manner, in stable on straw at the SGiCE-UAB from one week post-surgery until the end of the study. The light cycle was natural as well as the temperature and the humidity.

**Figure 7:**
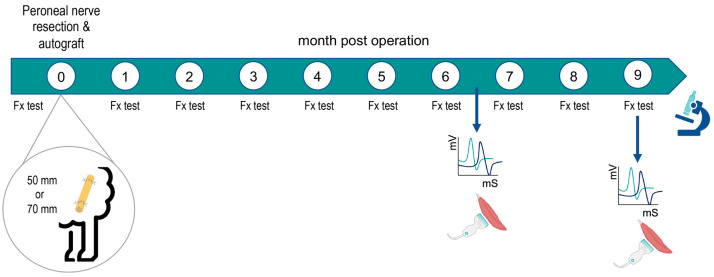
Schema of the design of the study. Following the surgery, functional tests (Fx test) were performed each month, electrophysiological tests and echography were made at 6.5 and 9 months, and samples were taken for histology at the end of the follow-up. Both operated and contralateral hindlimbs were tested at each interval.

Animals were divided in two experimental groups: autograft 5 cm (AG5; *n* = 4) and autograft 7 cm (AG7; *n* = 5) depending on the length of the graft used. Blood samples were taken and general health assessment was performed before inclusion in the study. Clinical signs, including general state, behavior, claudication, water and food intake, and wound healing during the observation period were assessed twice a day for 3 days after the surgery, once a day for one week, and then once a week until the end of the follow-up at 9 months post-surgery. In the operated and the contralateral (as control) hindlimbs, clinical evaluation of motor and sensory functions was performed prior to and at monthly intervals after surgery to evaluate nerve regeneration and reinnervation (Figure 7). Electrophysiological and echography testing was carried out at 6.5 and 9 mpo.

### 4.2. Surgical Procedure

Animals were fasted 16 h prior to the surgery to reduce the ruminal content and to prevent deviant swallowing and consequent risk of provoking aspiration. For the surgery, the sheep were sedated with an intramuscular injection of a mixture of midazolam (0.2 mg/kg) and morphine (0.4 mg/kg). A venous catheter was inserted in the cephalic vein and anesthesia was induced with propofol (4 mg/kg i.v.). Animals were intubated to maintain a proper anesthesia level by means of isoflurane (2 L/min) mixed with 100% oxygen. Fluid therapy was given with 10 mL/kg of Ringer solution, and a preoperative antibiotic dose of cefazoline (22 mg/kg) was administered intravenously. A gastric catheter was also placed in the stomach to avoid reflux during anesthesia. 

**Figure 8:**
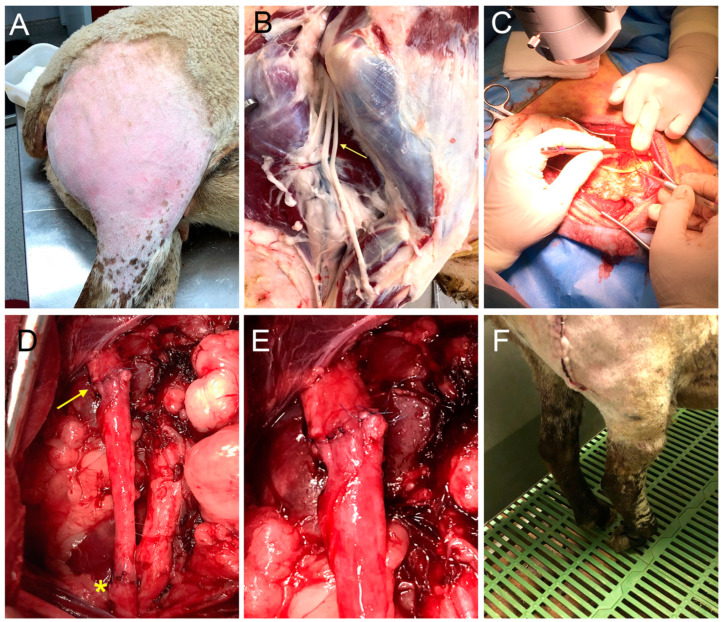
Photographs of the surgical procedure for the peroneal nerve injury and repair. (**A**) The surgical approach was performed with the animal in lateral recumbency through a lateral longitudinal skin incision. (**B**) Wide dissection showing the peroneal nerve location (arrow) after the sciatic nerve bifurcation into the tibial and peroneal nerve in a cadaveric sheep. (**C**) Resection of the common peroneal nerve under the operating microscope to create the nerve gap. (**D**) A 5 cm autograft was sutured again to the nerve stumps with epineural sutures (proximal suture marked with yellow arrow and distal suture marked with a yellow asterisk). (**E**) Detail of the 8 stitches made to join the nerve graft with the healthy nerve stump without tension. (**F**) After the surgery, some animals showed foot drop in the standing position.

The sheep was placed in a lateral decubitus position and the incision zone was shaved and cleaned with chlorhexidine solution. The peroneal nerve was exposed using a longitudinal lateral skin incision along the right thigh followed by splitting the semitendinosus and biceps femoris muscles. Under the operating microscope, a length of 50 or 70 mm of the common peroneal nerve was resected 1 cm above the iliac vein to create a nerve gap (Figure 8). The distance between the proximal cut of the peroneal nerve and the entrance of the distal nerve into the TA muscle was 34–36 cm. The two nerve stumps were then bridged with the resected nerve segment in the same orientation by means of 8/0 epineural sutures. The resistance of the coaptation was tested by slightly stretching the nerve. The incision was closed by layers and disinfected with povidone iodine solution.

After the surgery, the animals were transferred to the SGiCE-UAB, where they were housed in couples for one week in a controlled enclosure, and thereafter housed in groups at the regular sheep barn. Postoperative care included buprenorphine (0.01 mg/kg s.c.) twice a day for 2 days and meloxicam (0.2 mg/kg s.c.) once daily for 3 days. Long-term antibiotic ceftiofur (5 mg/kg s.c.) was administered after the surgery.

### 4.3. Functional Testing

**Figure 9:**
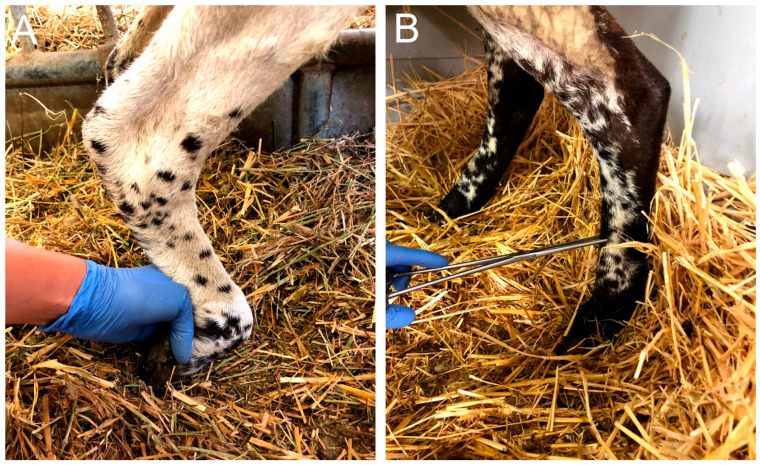
Photograph of the maneuver used for the proprioception test, assessing the capability of dorsiflexion of the hindfoot from a forced plantar flexion position (**A**). The flexor withdrawal reflex assesses the response to pinching the dorsum of the foot with a hemostat in a proximal point, a middle point (**B**) and a distal point.

Prior to the surgery and afterwards at monthly intervals, animals were tested for functional evidence of regeneration and reinnervation of the operated hindlimb compared with the contralateral hindlimb (Figure 7). Each parameter assessed was scored on a semiquantitative scale of 0 (no deficit), −1 (partial deficit or functional loss), or −2 (complete loss of response to the maneuver). In all the sessions, each functional test was validated by first performing the test on the non-operated control hindlimb of the same animal. Ankle and foot placement were assessed in the orthostatic position. Locomotion was evaluated during free walking in the pen and during fast walking to assess the ability to maintain plantar support and paying particular attention to the foot drop position of the operated hindlimb (0 = normal walk and maintenance of the plantar support; −1 = few failures on the plantar support maintenance; −2 = foot drop in most of the steps). The mass of the TA muscle was assessed by manual palpation comparing the contralateral hindlimb with the operated one (0 = similar mass; −1 = slight reduction; −2 = large reduction). The proprioceptive response was tested by the ability to replace the hoof from a forced plantar flexion position to a plantar support three times (0 = consistent response; −1 = one failure; −2 = two or three failures). The hindfoot withdrawal reflex was tested by assessing whether the animal withdrew the hindlimb when pinching with a forceps in three areas of the dorsal area of the hindfoot, proximal, middle, and distal (Figure 9). Each site was tested two different times and the score was independent between the three zones (0 = fast and brisk withdrawal response of the limb; −1 = weak or non-consistent response in 2 trials; −2 = no response in 2 trials. Two researchers, who were blind regarding the group allocated throughout the study period, scored on a 0–1–2 scale each of the above maneuvers for all the animals and test days; the score given was by agreement between the observers (Table 1).

### 4.4. Electrophysiological Tests

**Figure 10:**
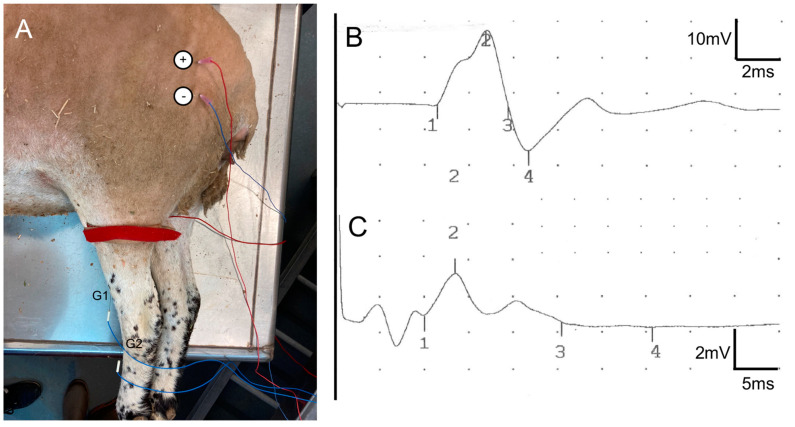
(**A**) Setup used for the nerve conduction studies. Stimulating needle electrodes were placed at the sciatic notch to stimulate the nerve (+–−), whereas recording needle electrodes were placed at the TA muscle (active electrode, G1) and at the distal tendon (reference, G2). The red band corresponds to the ground electrode. (**B**,**C**) Sample EMG recordings at 9 mpo. Top trace (**B**) shows the CMAP in the control hindlimb, and the bottom trace (**C**) in the operated hindlimb of a sheep of AG7 group. The onset of the CMAP is labeled with mark 1, the negative peak of the CMAP with mark 2, and the end with mark 3. Note the differences in time and voltage scales, noted at the right of each trace.

Electrophysiological tests to evaluate reinnervation of the TA muscle were performed at 6.5 and 9 mpo under general anesthesia. Animals were sedated with intravenous diazepam (0.25 mg/kg), and then anesthesia was induced with an intravenous injection of diazepam and ketamine (0.25 mg/kg and 5 mg/kg, respectively). The sciatic nerve was stimulated with transcutaneous needle electrodes placed at the sciatic notch using an EMG apparatus (Sapphire 4ME Medelec, Vickers Healthcare Co., Surrey, UK). The compound muscle action potential (CMAP) of the TA muscle was recorded with monopolar needle electrodes (Figure 10) from the control hindlimb and the operated hindlimb; the amplitude and onset latency were measured from the maximal response obtained. The stimulus intensity was progressively increased until a maximal amplitude CMAP was obtained, and the recorded CMAP corresponded with contraction of the TA muscle and hindlimb movement. In addition, free-running EMG recordings were made to detect fibrillation potentials as a sign of muscle denervation.

### 4.5. Ultrasound Test

**Figure 11:**
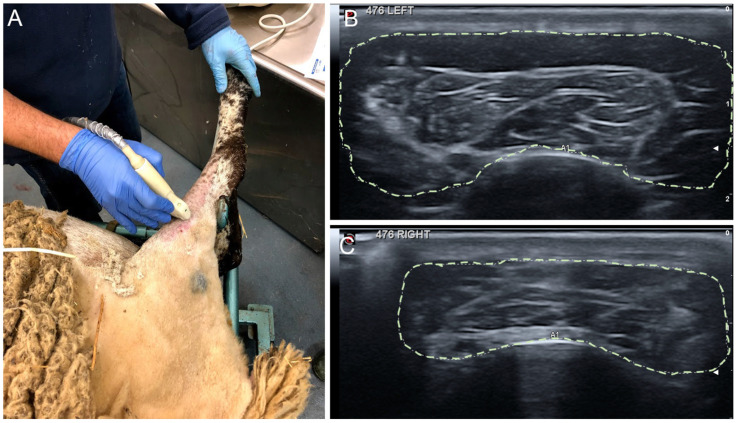
Procedure for the echographic imaging. The ultrasound probe was placed with conductive gel on the mid of the TA muscle mass (**A**). Sample images of the echography of TA muscle, delineated by discontinuous line in a control hindlimb (**B**), and in an operated hindlimb (**C**).

Echographic evaluation of the leg anterior compartment was performed with a MyLab^®^ Gamma apparatus (Esaote, Genova, Italy) at the same time of the electrophysiological tests, 6.5 and 9 mpo, under general anesthesia. Hair of the dorsal area of the leg was shaved, and the skin was cleaned with water and mild soap. To optimize image acquisition, acoustic gel was used. The size of the TA muscle, calculated as area and perimeter, was determined using the B-mode ultrasound (15 MHz), with a linear ultrasound probe in the cranial aspect of the crus at the midpoint between the tibial crest and the tuber calcanei (Figure 11). The control hindlimb of all animals was used as a control.

### 4.6. Histological Evaluation

At the end of the follow-up at 9 mpo, following electrophysiological and ultrasound tests and under general anesthesia, the sheep were euthanized with an intravenous administration of Euthasol (400 mg/kg). The sciatic nerve and branches were exposed under surgical dissection. The nerve graft, including the proximal and distal suture lines, was harvested, and fixed in 4% paraformaldehyde for 5 days at 4 °C. The TA muscle was dissected and weighted. Samples from the TA muscle and a piece of skin of the dorsum of the hindfoot were taken and fixed in paraformaldehyde 4% for 7 days at room temperature (RT). Samples from the contralateral hindlimb were taken as control samples.

The nerve graft was divided into different segments to analyze the middle segment of the graft and the nerve distal to the graft. Each segment was also divided in two halves. The first half of the nerve graft of section 2 and section 4, corresponding to the middle and distal to the nerve graft, respectively, were transferred to 70° ethanol for 48 h. Samples were embedded in paraffin and 5 mm thick cross-sections were cut on a microtome. Some slides were deparaffinated and stained with hematoxylin and eosin to visualize the general structure of the nerve graft under light microscopy. Other slides were processed for immunohistochemistry. The latter slides were deparaffinated and blocked with a solution of normal goat serum and normal donkey serum (10%) in phosphate-buffered solution (PBS) containing 0.3% Triton. The sections were incubated overnight at RT with primary antibodies against neurofilament (NF200; myelinated axons; 1:400; AB5539-Millipore) and against S100 protein (S100; Schwann Cells; 1:50; 22520-DiaSorin). Following washes, sections were incubated against secondary antibodies bound to Alexa Fluor 488 and Alexa Fluor 594. Immunolabeled sections were viewed under epifluorescence microscopy (Olympus BX51). The total number of regenerated myelinated axons was estimated by measuring the cross-sectional area of the nerve grafts and counting myelinated axons labeled with NF200 in selected fields distal to the nerve grafts.

The second half of the sections were post-fixed in 3% paraformaldehyde and 3% glutaraldehyde in cacodylate-budder solution (0.1 m, pH 7.4) at 4 °C. The samples were post-fixed in osmium tetroxide (2% for 2 h), dehydrated with ethanol, and embedded in Epon resin. Semithin sections 0.5 μm thick were cut on an ultramicrotome (Leica, Wetzlar, Germany) and stained with toluidine blue. Representative light microscopic images were selected.

Samples from TA muscle and skin of the dorsum of the hindfoot were embedded in paraffin, sectioned, and stained with hematoxylin and eosin to visualize the general structure.

### 4.7. Data Analysis

All the data are expressed as mean ± standard error of the mean (SEM). Statistical comparisons of functional and histology results were analyzed by using Student’s *t* test and two-way ANOVA after testing for normal distribution. The GraphPad Prism 9 software was used for analysis and graphic representations. Statistical significance was considered if *p* < 0.05.

## Figures and Tables

**Table 1:** Items used in the functional evaluation of sheep after injury and repair of the peroneal nerve. The score points negative values in the case of deficit or loss of response.

Parameter	0	−1	−2
Locomotion	Normal gait	Limp	Drag the limb
Muscle loss (TA)	No loss	Reduced	Atrophy
Proprioception	Present	Decreased	Absent
Flexor withdrawal reflex (proximal point)	Present	Decreased	Absent
Flexor withdrawal reflex (middle point)	Present	Decreased	Absent
Flexor withdrawal reflex (distal point)	Present	Decreased	Absent

## Data Availability

The data presented in this study are available on reasonable request from the corresponding author.
